# *Rhizoglomus variabile* and *Nanoglomus plukenetiae,* Native to Peru, Promote Coffee Growth in Western Amazonia

**DOI:** 10.3390/microorganisms11122883

**Published:** 2023-11-29

**Authors:** Mike Anderson Corazon-Guivin, Gabriel Romero-Cachique, Karen M. Del Aguila, Amner Padilla-Domínguez, Angel David Hernández-Amasifuen, Agustin Cerna-Mendoza, Danny Coyne, Fritz Oehl

**Affiliations:** 1 Laboratorio de Biología y Genética Molecular, Universidad Nacional de San Martín, Jr. Amorarca N° 315, Morales 22201, Peru; gabrielromerc@gmail.com (G.R.-C.); marilu.parillo@outlook.com (K.M.D.A.); amnerpadilla701@gmail.com (A.P.-D.); ahernandeza@unsm.edu.pe (A.D.H.-A.); acerna@unsm.edu.pe (A.C.-M.); 2 International Institute of Tropical Agriculture (IITA), Headquarters PMB 5320, Oyo Road, Ibadan 200001, Oyo State, Nigeria; d.coyne@cgiar.org; 3 Agroscope, Competence Division for Plants and Plant Products, Plant Protection Products—Impact and Assessment, Applied Ecotoxicology, Müller-Thurgau-Strasse 29, 8820 Wädenswil, Switzerland

**Keywords:** beneficial fungi, bio-fertilizer, biological agents, crops, Glomeraceae, Glomeromycota, inoculation, sustainable agriculture

## Abstract

Coffee (*Coffea arabica*) is among the world’s most economically important crops. Coffee was shown to be highly dependent on arbuscular mycorrhizal fungi (AMF) in traditionally managed coffee plantations in the tropics. The objective of this study was to assess AMF species richness in coffee plantations of four provinces in Perú, to isolate AMF isolates native to these provinces, and to test the effects of selected indigenous AMF strains on coffee growth. AMF species were identified by morphological tools on the genus level, and if possible further to the species level. Two native species, *Rhizoglomus variabile* and *Nanoglomus plukenetiae*, recently described from the Peruvian mountain ranges, were successfully cultured in the greenhouse on host plants. In two independent experiments, both species were assessed for their ability to colonize coffee seedlings and improve coffee growth over 135 days. A total of 35 AMF morphospecies were identified from 12 plantations. The two inoculated species effectively colonized coffee roots, which resulted in 3.0–8.6 times higher shoot, root and total biomass, when compared to the non-mycorrhizal controls. *R. variabile* was superior to *N. plukenetiae* in all measured parameters, increasing shoot, root, and total biomass dry weight by 4.7, 8.6 and 5.5 times, respectively. The dual inoculation of both species, however, did not further improve plant growth, when compared to single-species inoculations. The colonization of coffee by either *R. variabile* or *N. plukenetiae* strongly enhances coffee plant growth. *R. variabile*, in particular, offers enormous potential for improving coffee establishment and productivity. Assessment of further AMF species, including species from other AMF families should be considered for optimization of coffee growth promotion, both alone and in combination with *R. variabile*.

## 1. Introduction

Coffee (*Coffea arabica* L.) is highly valued for its flavor, fragrance and caffeine content, making it one of the most consumed agricultural commodities worldwide with significant economic and social implications [1]. It is cultivated across the tropics and sub-tropics both on industrial-scale commercial plantations as well as family-owned smallholder plots [2,3]. In South America, Perú is an important producer of coffee and is considered among the ten most important coffee-producing countries globally. Annual exports are currently in the range of 210,000 tons per annum, with marked increases in recent years, representing an annual income of 1.1 billion dollars for the more than 200,000 families of small farmers involved in its production [4,5]. Within Perú, the San Martín and neighboring Amazonia Regions account for approximately 50% of the national coffee production [6].

Worldwide, coffee has traditionally been cultivated under the shade-green system, but with a steady rise in the worldwide demand for coffee, production systems have increasingly shifted to more intensive full-sun systems [7,8]. As a consequence, the majority of coffee plantations are no longer shade-grown, which accounts for approximately 25% of the total production area [8]. However, in Central and South America, shade-grown coffee remains a dominant production system, with the notable exceptions of Brazil and Colombia [8]. In Perú, the majority of coffee farms (‘fincas cafeteleras’) are highly biodiverse with relatively high levels of shade, and cultivation practices characterized by low reliance on agrochemicals [6,8,9]. Most Perúvian coffee growers though, irrespective of the cropping system, rely on some type of organic-based mineral fertilizer to improve crop performance [9]. Perú stands as a principal exporter of ‘organic coffee’ [6], where the cropping systems promote a slower and more equilibrated development of the coffee-cherries or beans [10], which creates a superior organoleptic quality [11,12], increasing its value in the ‘specialty coffee’ market [10,13]. Over recent years, the market for ‘specialty coffee’ has risen, with indications for this to continue increasing [8,12], mainly benefiting smallholder farmers, who represent around 70% of coffee producers worldwide [14].

In organic and biologically-based coffee production systems, the preservation of diverse microbial soil communities towards the maintenance of soil health is a key component for ecosystem stability and multifunctionality [15,16,17]. For example, a reduced soil microbial diversity in coffee monocultures negatively affected several soil functions, suppressing plant growth and reducing coffee production [18]. Additionally, a higher microbiome diversity is also associated with better host plant suppression of diseases and enhanced growth on various crops [19,20]. Within these microbial communities, arbuscular mycorrhizal fungi (herein abbreviated as AM fungi or AMF) (phylum Glomeromycota) are key components [19,21,22], being associated with 70–90% of plant species in terrestrial ecosystems [23] and supporting significantly improved plant growth through their extensive extraradical networks and effective soil nutrient acquisition (P, N, S, K and several microelements [24,25,26,27,28,29,30,31,32,33]. Inoculation of AM fungi can be highly beneficial to crop production [34,35]. Establishing whether single or mixed species applications are more advantageous, however, needs to be assessed for each target crop under prevailing conditions as it is often not initially clear if inoculation with one species is superior to multi-species inoculation [36,37,38,39,40,41]. Berruti et al. [42] concluded that the three globally prevalent AMF species, *Rhizoglomus intraradices*, *Funneliformis mosseae,* and *R. irregulare*, all belonging to the family Glomeraceae, are the most popularly used inocula. These species are widespread around the globe, colonize a large majority of plant species, and are adapted to a large spectrum of edaphoclimatic conditions [43]. Otherwise, it might be more advantageous to use native, and thus more adapted AMF strains, both for ecological-climatological and agronomic reasons [44].

Coffee plants naturally form AMF associations [45,46], showing a high mycotrophy from the early seedling stage to more advanced, mature stages [47,48,49,50]. Various studies have determined a high AMF diversity in the coffee rhizosphere [51,52], and that the application of AMF as biofertilizers improves seedling development and plant growth in the greenhouse [48,50,53,54,55,56]. In total, 70 AMF species have been recorded from the coffee rhizosphere globally [57], but this is most likely a gross underestimation since comprehensive studies to assess AMF diversity in coffee have only recently been undertaken, mostly within the last two decades. To date, just ~330 AMF species have been described [58], with estimates indicating that over 1500–2000 AMF species exist [59,60]. Within Perú, recent studies have led to the description of several new AMF species, such as from the rhizosphere of inka nut (*Plukenetia volubilis*), cocoa (*Theobroma cacao*) as well as from coffee [61,62,63,64,65,66]. Studies have also shown that native AMF strains or inocula originating from coffee plantations are more suitable and provide greater benefits to coffee than the commercial, bulk-produced strains/species from abroad [67], an effect similarly observed for other crops [42,44]. In this context, the present study was undertaken to evaluate the AMF diversity in coffee plantations in the San Martín State of Perú and whether single or combined inoculations of prevalent native species provided better biofertilizer effects on seedling development and early coffee growth. We hypothesized that a high AMF diversity can be found in Peruvian coffee plantations and that the combined inoculation of native AMF species, belonging to the Glomeraceae, would be superior for coffee growth than the inoculation with only one AMF species.

## 2. Materials and Methods

### 2.1. Coffee Plantations under Study

Between March and April 2016, soil samples (0–20 cm depth) were taken from the coffee rhizosphere of 12 coffee plantations (sites) located in 4 provinces of San Martín State (Table 1). For each site, five plants were selected randomly and sampled separately, with ~1 kg of rhizospheric soil extracted from four equidistant points around the main stem of each plant. Each soil sample was placed in a single plastic bag, constituting five samples in total per site, i.e., five replicates per site. The soil samples were transferred to the laboratory within one day, sieved (5 mm size), air-dried and stored at 4 °C.

### 2.2. AMF Spore Isolation, Identification and Diversity

The AMF was extracted by wet sieving from 50 g soil according to Gerdemann and Nicolson [68] using 38 and 250 μm sieves, followed by a sucrose density gradient centrifugation as described by Sieverding [69]. Species were morphologically identified and counted as described below, and those species with abundant and ubiquitous occurrence (90–100%) were selected for further multiplication. Species occurrence was calculated from the number of sites each species was detected, divided by the total number of sites investigated [70].

Spores were observed under a compound microscope after mounting in polyvinyl alcohol-lactic acid-glycerol (PVLG) [71], Melzer’s reagent, a mixture of PVLG and Melzer’s reagent [72], a 1:1 mixture of lactic acid and water, and in water [73]. The AMF taxa were identified on the genus level, and, if possible, up to the species level, using described morphological spore characteristics, type of spore formation, and their sub-cellular structures, such as their color, size, number, and structure of walls and wall layers [74,75] using the Glomeromycota system as presented by Wijayawardene et al. [58] and updated by Blaszkowski et al. (2022) [76] and da Silva et al. [77].

The AMF spore abundance and species richness at the 12 sampling sites were determined by counting the number of spores identified by species and counting the number of species detected per site, respectively. From each field site a detailed list of AM fungal species was developed, classifying the spore abundance into four categories (0, 1–2, 3–5 and ≥6 spores per g soil). The most abundant and frequently occurring species were selected for culturing and further assessment.

### 2.3. Multiplication of Selected AMF Species

The most abundant and ubiquitous species from the field soil sample evaluation were multiplied individually using a polyculture of *Sorghum vulgare*, *Brachiaria brizantha* and *Medicago sativa*. For the multiplication, all 12 field soils were used in 2.5 L pots (~2 kg soil per pot). Each field soil was first mixed with sand (2:1, *v*/*v*) before autoclaving at 121 °C for 1 h per day over three days. Five pots per site were used for the cultivation of each AMF species. The pots were maintained in the greenhouse under ambient temperature conditions (Temperature oscillated between 21.4 and 33.2 °C, and relative humidity between 48 and 75%) for three months before harvesting of the AM fungal inocula. The AM fungal species that successfully multiplied were used as inocula in two subsequent experiments, to study their potential to improve coffee plant growth in the greenhouse. These were *Rhizoglomus variabile* and *Nanoglomus plukenetiae* (Corazon-Guivin et al. [62,64]), recently described from the study region and firstly isolated from the rhizosphere of *Plukenetia volubilis* L., an indigenous agroforestry crop of increasing agronomic importance. Both AMF species had also been analyzed phylogenetically to confirm the morphological identification of these AM fungal species (see Corazon-Guivin et al. [62,64]). The two AMF strains used for the present study were isolated from a coffee plantation in Pamashto (*R. variabile*) and in Pueblo Nuevo (*N. plukenetiae*; Table 1).

### 2.4. Effects of AMF Inoculation on Coffee Growth: Experimental Details

The effects of the two species, *R. variabile* and *N. plukenetiae*, were assessed on coffee crop growth in the greenhouse of the Laboratorio de Biología y Genética Molecular, Universidad Nacional de San Martín (Distrito Morales, Jr. Amorarca, cdra. 3 s/n), located in the province of San Martín in San Martín State (06°35’28’’ S, 76°18’47’’ W) at 230 m a.s.l. altitude. The experiment was conducted between March and July 2018 and repeated between May and September 2018. The pots were maintained under greenhouse conditions at a mean daily temperature of 29 °C (maximum of 38.2 °C, minimum of 21.4 °C). Mean, maximum and minimum relative humidity were 64.0, 73.8 and 47.9%, respectively during the period March to September 2018.

**Figure 1:**
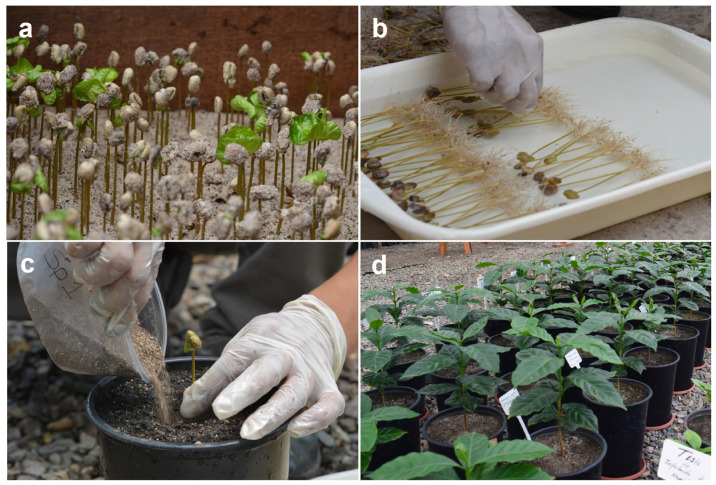
Inoculation process of coffee seedlings with arbuscular mycorrhizal fungi (AMF), (**a**) Growing coffee seeds in nursery beds, (**b**) Uniform seedlings at the “little soldier” stage, (**c**) Inoculation and transplantation of coffee seedlings, (**d**) Vegetative growth of coffee plants.

In both experiments, *C. arabica* cv. Caturra plants aged aprox. one month was used, which is among the most common cultivars cultivated in Perú. Ripe red coffee cherries were selectively collected from healthy plants, without any obvious pest or disease symptoms, from a field in “Naranjal” ubicate in the Rioja Province of the San Martín Department, Perú in 2018. The berries were manually de-pulped, discarding any small-sized seeds and dried under shade. They were then surface sterilized by dipping in 0.5% sodium hypochlorite for 2 min and 95% ethanol for 2 min, and then rinsed in sterile distilled water three times before placing in germination boxes (1 × 1 × 0.3 m), using autoclaved (121 °C, 15 p.s.i., 30 min) coarse sand as a substrate. The seeds were placed flat-side down onto the sand, spaced 2.5–3.0 cm in regular rows and covered with a thin layer of finely sieved coarse sand (2 mm mesh width). After sowing, the seed beds were mulched with a mesh raschel (80%) to protect the seeds from desiccation and create optimal temperatures of ~25 °C for seed germination. The seed beds were irrigated daily over several weeks until sufficient uniform plants at the “little soldier” growth stage were available (~15 cm high), soon after emergence and before the seed-coat was cast off at ~20 days after emergence (Figure 1a,b).

Field soil mixed with coarse river sand (2:1, *v*/*v*) was used after autoclaving for 1 h per day over three consecutive days. The textural classification of this substrate was a sandy-loam, with 4.82 of pH, 0.35 dSm^−1^ electrical conductivity, 1.66% organic matter, 6.5 mg P kg^−1^, and 63 mg K kg^−1^ (0.14 K^+^meq/100 g). The uniform “little soldier” seedlings were bare-root transplanted singularly into plastic 3 L pots, filled with 3 kg of sterile substrate. The pots were first disinfected with ethanol and rinsed with distilled water. The experiment comprised 4 treatments, each with 36 replications, arranged in a completely randomized design totaling 144 seedlings, which were placed on greenhouse tables. Experimental treatments included: (1) single inoculation of *R. variabile* (Rv) and of (2) *N. plukenetiae* (Np), (3) combined inoculation of *R. variabile* and *N. plukenetiae* (Rv+Np), (4) non-mycorrhizal control (Ctrl).

In both experiments, *R. variabile* and *N. plukenetiae* were inoculated using 20 g freshly chopped pieces of mycorrhizal roots from *S. vulgare*, *B. brizantha* and *M. sativa*, which included hyphae and ~1500 AMF spores; for dual inoculation (Rv+Np), 10 g chopped roots each of the two inocula were mixed and inoculated (Figure 1c). The non-mycorrhizal control treatment received washings of 20 g of the inoculum mixture filtered through Whatman n°. 42 filter paper. Inoculation of the AMF was conducted at transplanting. Holes 10 cm depth and 4 cm diameter were prepared using a trowel, which were first filled with inocula before placing plantlets directly onto the inocula and then completely filling with sterile soil substrate. The soil moisture was increased to maximum water-holding capacity and plants subsequently irrigated every three days to maintain the substrate at field capacity. Fertilizer was applied weekly using 75 mL of the Long Ashton nutrient solution [78], modified to supply 10.25 µg P mL^−1^ pot.

### 2.5. Assessment of Plant Characteristics

Plant height (cm), stem diameter (mm) and number of leaves were measured at 30 days after transplanting and then at 15-day intervals until harvest at 135 days. Chlorophyll content (SPAD) was also recorded on the youngest completely expanded leaf of each plant using a chlorophyll analyzer (SPAD-502, Minolta Camera Co. Ltd., Osaka, Japan). The leaf area (cm^2^) was calculated using ImageJ (https://imagej.nih.gov/ij/). At harvest, the plant’s total fresh weight was determined using a balance (OHAUS, Adventurer™ Parsippany, NJ, USA) and the total dry matter was recorded after oven drying at 80 °C for 48 h.

### 2.6. Arbuscular Mycorrhizal Root Colonization

The freshly harvested roots were rinsed with tap water, and the percentage of mycorrhizal colonization was estimated using the root clearing and staining method [79], with modifications. Once stained, roots were cut into 1 cm segments, mounted on slides and observed under a compound microscope (NIKON, Eclipse E200, Tokyo, Japan) at 20× magnification [80].

### 2.7. Analyses of N, P and K Coffee Plant Contents

At harvest, plant N, P and K contents were analyzed using total leaf matter from each plant. The N concentration was obtained using the Kjeldahl method [81], P concentration following digestion in HNO_3_:HClO_4_ (4:1), spectr. UV-Vis (λ = 420 nm) and K concentration by digestion in HNO_3_:HClO_4_ (4:1) for atomic absorption spectrophotometry analyses (Model Varian, AAS Spectra 55B, Victoria, Australia).

### 2.8. Statistical Analyses

The results of the two independent experiments showed only minor numerical differences, but no statistical differences (*p* > 0.05) for each parameter recorded. Thus, the data for the two experiments were combined for analysis. All measured variables were evaluated for normality and homogeneity using Shapiro–Wilk [82] and Levene’s [83] tests, respectively. All the variables evaluated were transformed to a natural logarithm (ln), except for dry biomass and colonization, which were transformed to a square root (√ x) to normalize data. ANOVA analyses were followed by Tukey’s HSD to test for differences among treatments at *p* < 0.05 significance level [84]. The data were analyzed using INFOSTAT version 2012.1 software [85]. The ANOVA and the mean comparison tests were conducted on transformed data, with data back-transformed to the original units for presentation in results.

## 3. Results

### 3.1. AMF Species Richness and Spore Abundance per Species

In total, 35 AMF morphospecies belonging to 13 genera, were identified from the 12 study sites by morphological spore identification (Table 2). Of the 35 species, eleven were not unequivocally identified on the species level. Three of them resembledknown species, while the lasting eight species might be new to science, according to our knowledge, and will be part of future taxonomic analyses. At individual sites, between 6 and 18 species were recorded. Three AMF species were detected across all sites (*G. microcarpum*, *R. variabile* and *N. plukenetiae*), while *A. mellea* was recovered from 11 sites, and *G. brohultii* from 10 sites. Five further species were found in at least six sites but with lower abundance than the five most frequently occurring species. The remaining 25 morphospecies were less abundantly and less frequently observed, or only sporadically detected (Table 2).

### 3.2. AMF Species Selected for Further Multiplication and Functional Experiments

The species *G. microcarpum*, *R. variabile*, *N. plukenetiae* and *A. mellea* were the most abundant and most frequently occurring (92–100% of sites; Table 2). Following three months of culturing in the greenhouse on *S. vulgare*, *B. brizantha* and *M. sativa* in the twelve soils, *R. variabile* and *N. plukenetiae* were the only species that multiplied well and with a high spore abundance (Table 3), while *A. mellea* and *G. microcarpum* did not multiply well. Therefore, *R. variabile* and *N. plukenetiae* only were considered for further functional experiments on coffee crop growth promotion.

### 3.3. Effects of AMF on Coffee Plant Growth

**Figure 2:**
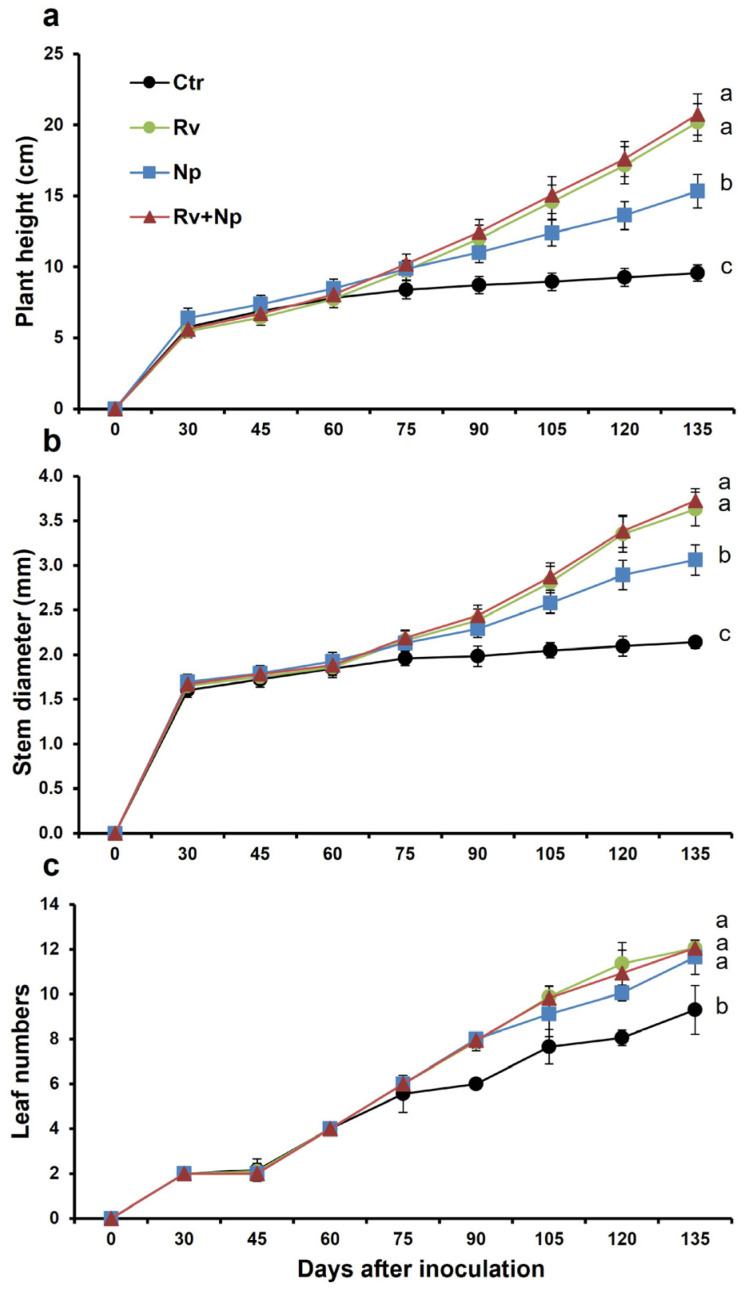
Effect of single and dual arbuscular mycorrhizal fungal inoculation on (**a**) plant height (cm), (**b**) number of coffee leaves per plant and (**c**) stem diameter (cm) in coffee plants along 135 days of vegetative growth measured (15-day intervals). Mean values per treatment. Error bars indicate standard deviation (±S.D.). Different letters indicate significantly different means (*p* < 0.05). Ctr = Non-inoculated, Rv = Inoculation with *Rhizoglomus variabile*, Np = Inoculation with *Nanoglomus plukenetiae*.

**Figure 3:**
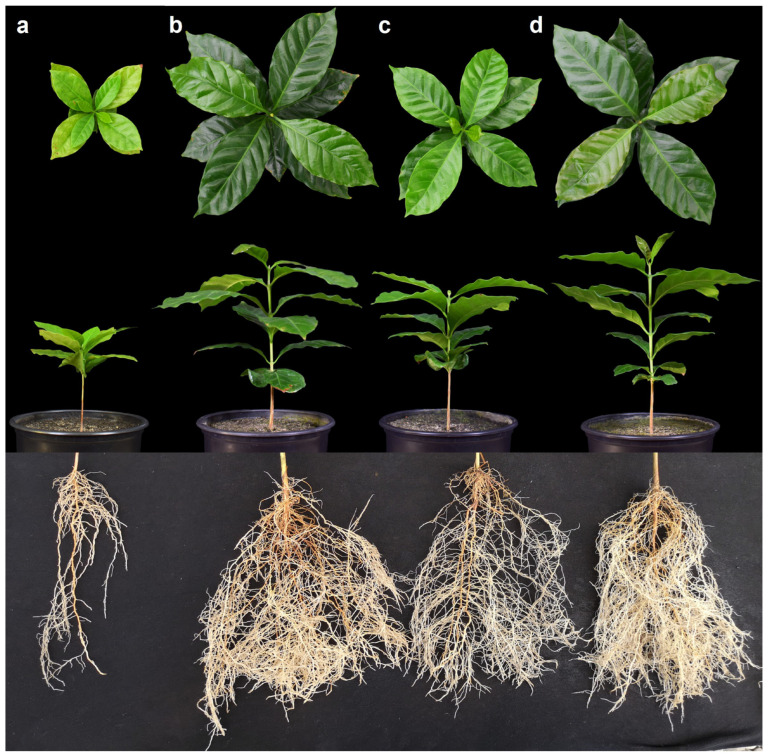
Illustration of coffee leaf, shoot and root growth 135 days in (**a**) non-mycorrhizal control plants, after single inoculation with (**b**) *Rhizoglomus variabile*, or (**c**) *Nanoglomus plukenetiae*, and (**d**) after dual inoculation with both AMF species.

The plant growth parameters began to differentiate between treatments at 60–75 days after inoculation, with mycorrhizal plants having better growth than the controls (Figure 2). At harvest, mycorrhizal plants were taller and had more leaves and thicker stems than non-mycorrhizal plants. Plants dual-inoculated and with *R. variabile* alone were tallest (mean plant height 20.7 and 20.2 cm, respectively), and 2.2 times higher (*p* < 0.05) than plants inoculated with *N. plukenetiae* alone (15.3 cm; 1.5 times higher than control plants), and the non-mycorrhizal control (9.6 cm; Figure 3a, F = 655.38, *p* < 0.0001). Similarly, stem diameter was higher in the dual-inoculated (3.7 mm) and *R. variabile*-inoculated (3.6 mm) plants than the *N. plukenetiae*-inoculated (3.1 mm), or in the non-mycorrhizal control (2.1 mm), with mycorrhizal stem girths 1.4–1.7 times thicker than non-mycorrhizal plants (Figure 3b, F = 1061.76, *p* < 0.0001). All AMF-treated plants had more leaves than the control (Figure 3c, F = 118.86, *p* < 0.0001), with approximately 1.3 times more leaves than the non-mycorrhizal control at harvest. In line with leaf number, the leaf area index per plant was greatest in the dual-inoculated (709 cm^2^) and with *R. variabile*-inoculated (691 cm^2^) plants and significantly lower in *N. plukenetiae*-inoculated plants (434 cm^2^), and lowest in the non-mycorrhizal control plants (155 cm^2^), and thus up to 4.6 times higher in the mycorrhizal than non-mycorrhizal treatments (Table 4).

At harvest, inoculation of coffee seedlings with AMF led to significantly greater shoot and root growth (Table 4; Figure 3). Coffee plant fresh and dry shoot and root weights were consistently heavier after inoculation with dual AMF and single inoculation with *R. variabile* compared with inoculation of *N. plukenetiae* alone, and lowest in the non-mycorrhizal control (Table 4; Figure 3). The total coffee fresh plant weight was 6.2 and 6.0 times higher in the dual-inoculated and the *R. variabile*-inoculated treatments, respectively, compared to the non-mycorrhizal control plants, and total dry weight was 5.5 times heavier for the dual-inoculated plants.

The shoot fresh weight of coffee plants was 3.2–5.3 times higher in the mycorrhizal than non-mycorrhizal treatments (Table 4), and the root fresh weight of the mycorrhizal coffee plants was 5.5–9.8 times higher in the mycorrhizal treatments. The shoot dry weight of the mycorrhizal plants was 2.9–4.7 times higher in the mycorrhizal than non-mycorrhizal treatments, and the root dry weight of mycorrhizal plants was 5.0–8.6 times higher. Also, the leaf area index was highest after dual inoculation and inoculation with *R. variabile* alone (Leaf area 709 and 691 cm^2^ per plant, respectively), while it was significantly lower with *N. plukenetiae* alone (434 cm^2^), and lowest in the non-mycorrhizal control (155 cm^2^), meaning that the leaf area of mycorrhizal plants was 2.8–4.6 times higher in the mycorrhizal than non-mycorrhizal treatments (Table 4).

### 3.4. Arbuscular Mycorrhizal Root Colonization in the Growth-Response Experiments

**Figure 4:**
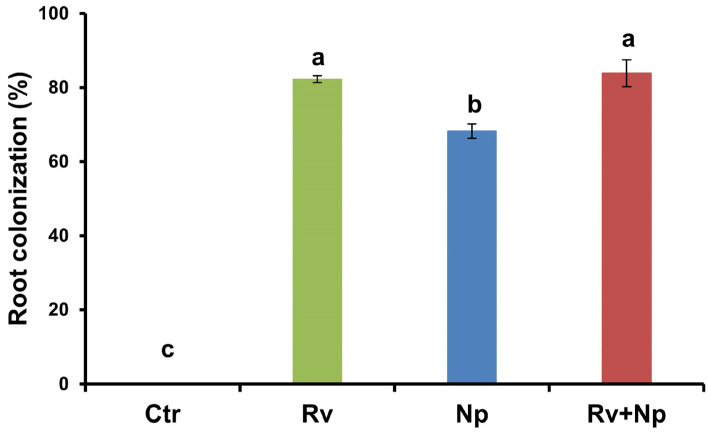
AM fungal root colonization of coffee plants inoculated with *Rhizoglomus variabile*, *Nanoglomus plukenetiae* or a combination in the greenhouse after 135 days. Mean values per treatment (N = 34). Error bars indicate standard deviation (±S.D.). Columns sharing the same letter were not significantly different (*p* < 0.05). Ctr = Non-inoculated, Rv = Inoculation with *Rhizoglomus variabile*, Np = Inoculation with *Nanoglomus plukenetiae*.

At harvest, all mycorrhizal treatments colonized roots to a high percentage (68–84%) by *R. variabile*, *N. plukenetiae,* or both fungi, while the non-mycorrhizal control was absent of any colonization. The highest (*p* < 0.05) colonization occurred with *R. variabile* (82%) and *R. variabile* + *N. plukenetiae* (84%), compared to *N. plukenetiae* alone (68%). See Figure 4, F = 4864.4, *p* < 0.0001.

### 3.5. Impact of AMF on Chlorophyll and Mineral Nutrient Contents in Coffee Leaves

**Figure 5:**
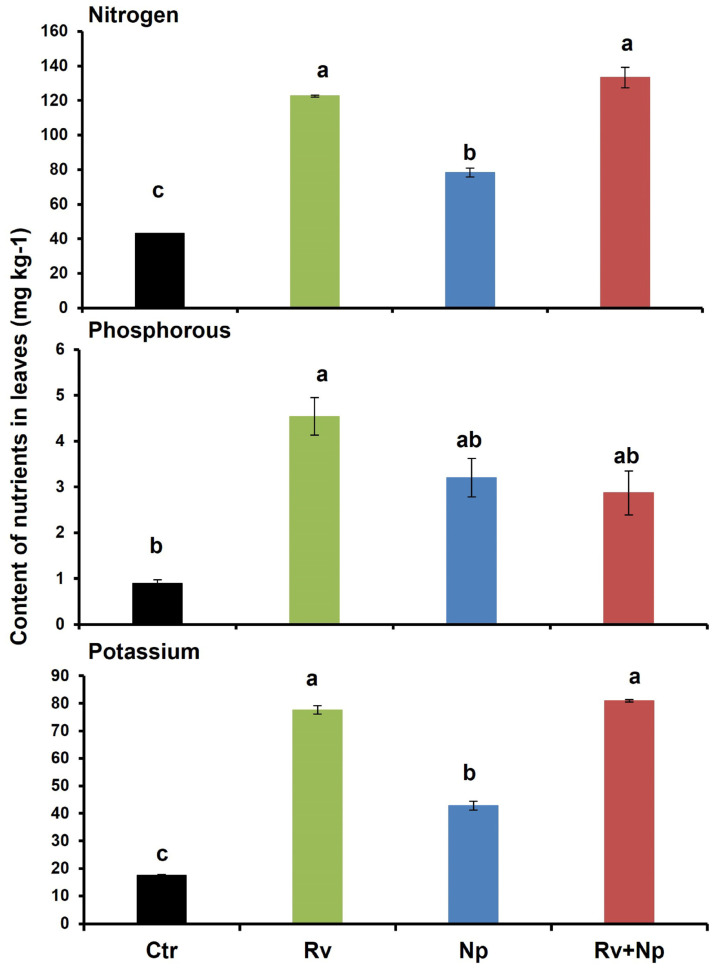
Nitrogen, phosphorus and potassium contents (mg kg^−1^) of coffee leaves after 135 days in non-mycorrhizal control plants (Ctr), after single inoculation with *Rhizoglomus variabile* (Rv), or *Nanoglomus plukenetiae* (Np), and after dual inoculation with both AMF species (Rv+Np). Mean values per treatment. Error bars indicate standard deviation (±S.D.). Columns sharing the same letter were not significantly different (*p* < 0.05). Ctr = Non-inoculated, Rv = Inoculation with *Rhizoglomus variabile*, Np = Inoculation with *Nanoglomus plukenetiae*.

The highest leaf chlorophyll content was recorded following inoculation with both AMF and with *R. variabile* alone (60.3 and 59.8, respectively), and significantly lower with *N. plukenetiae* alone (48.9), and the non-mycorrhizal control (27.4 cm^2^), which was less than half of the dual-inoculated and *R. variabile*-inoculated plants (Table 4). The leaf N, P and K contents were also significantly higher in plants following AMF inoculation (Figure 5, nitrogen: F = 55.40, *p* < 0.0001, phosphorus: F = 5.21, *p* < 0.027 and pottassium: F = 229.80, *p* < 0.0001) at 2.9, 5.0 and 4.4 times higher, respectively, when inoculated with *R. variabile* than in the non-mycorrhizal plants. For *N. plukenetiae*-inoculated plants, the differences were less pronounced than for *R. variabile* with 1.8, 3.6 and 2.5 times higher N, P and K than in the non-mycorrhizal plants. Following dual-inoculation with both fungi the effects were similar to those with *R. variabile* for N and K contents, with 3.0 and 4.6 times higher than in the non-mycorrhizal control, while P content was more similar to the P content with *N. plukenetiae* (3.2 times higher than non-mycorrhizal plants).

## 4. Discussion

AMF species can be identified from field soil samples either by morphological spore or molecular analyses after DNA extraction [86]. Both approaches have their limitations and their advantages as described in Oehl et al. [87], but both are laborious and thus time-consuming and costly. Ideally, they are concomitantly used and should deliver complementary data, but the combination of both has been rarely applied so far, also due to the lack of skills for one or the other methodology [88]. In one of these rare events, morphological AMF identification was even found to be superior to the molecular approach [89]. In this study, AMF taxa were identified on the genus level (e.g., *Glomus*) and wherever possible to further identify the morphospecies (e.g., *Glomus microcarpum*, when the morphotype was unequivocally attributable to this species) or to the morphotype level (e.g., *Glomus* sp. resembling *G. spinuliferum*, when the species resembled to a known species; or e.g., *Glomus* sp. 1, when the species most probably is new to science, according to our knowledge).

In the four coffee-growing provinces of Peruvian San Martin State, 35 different morphospecies were detected in 12 coffee plantations, with 14–24 morphospecies recovered per province. The majority of previous studies with coffee report a similar AMF species richness (16–37 species) as found in our study [90,91,92,93,94,95,96], with some reporting higher species richness (43 and 79 species; [52,97]). The large variation in AMF species richness was often explained by the coffee production systems, with a generally higher AMF richness under the shadow (shade-green systems), as these systems allow the interaction of coffee with other plant species of the plantations through the belowground AMF network, as well as by different climatic conditions of the regions and the edaphic soil characteristics [93].

In our study, the species belonged to 13 AMF genera from 6 families, which demonstrates a high diversity, when compared with most studies (already cited within this paragraph). *Acaulospora* and *Glomus* genera provided the highest species richness, with 9 and 10 AMF species recorded, respectively. These genera are often the most prevalent in coffee plantations [91,94,98,99]. The genera *Rhizoglomus*, *Sclerocystis*, *Entrophospora*, *Dominikia*, *Ambispora*, *Diversispora*, *Gigaspora* and *Sieverdingia* have also been reported from coffee plantations worldwide [90,91,93,94,95,96,97] but represent a lower species richness, as reflected in the current study.

The key important observation of this study was the impressive increase in coffee seedling growth and development, following inoculation with the two indigenous AMF species *R. variabile* and *N. plukenetiae*, resulting in up to 5.5 times higher total biomass after 135 days, when compared with non-inoculated plants. This is the first functional study with *R. variabile* and *N. plukenetiae*, which were only recently described from coffee plantations in San Martín State, Perú [62,64]. The inoculation of *R. variabile* alone improved coffee growth to a greater extent than that of *N. plukenetiae*. This is notable, with now a fourth species within the genus *Rhizoglomus*, besides the well-known species *R. intraradices* and *R. irregulare* [42], and *R. invermaium* [100], with elevated beneficial effects on an agronomically important crop, underlining the importance of *Rhizoglomus* species for crop plant nutrition. The results clearly demonstrate the highly mycotrophic nature of coffee, as evidenced by other AMF-coffee associations [48,50,53,56,101] but moreover, highlight the outstanding functions of *Rhizoglomus* for plant nutrition with now a new ‘super strain candidate’ *R. variabile* for South American coffee plant establishment. Also, during a study to assess more than 40 AMF strains for their effect on leek (*Allium porrum*) growth, *Rhizoglomus* species and the closely related *Oehlia diaphana*, were the most beneficial species detected [100].

Our results demonstrate that the genus *Nanoglomus* (*N. plukenetiae*) from the family Glomeraceae can be added to those that provide strong beneficial effects on plant growth. *Nanoglomus plukenetiae* belongs to the major *Dominikia* clade of the Glomeraceae, which has previously not been recognized among the most beneficial clades of AMF [100].

The combination of both AMF inoculants did not lead to increased coffee growth, over inoculation with *R. variabile* alone. The effects of multiple species inoculations do not necessarily lead to improvements over single inoculations [48,101,102], although Trejo et al. [50] reported significant coffee growth improvement with various combinations of AMF consortia. More research and information is needed if, or in which cases, or for which crop species, single or multiple inoculations with specific AMF species/strains should be recommended. This would also likely depend on crop cultivar, as well as on the prevailing edaphic and climatic conditions, and whether cultivated in mono- or mixed cropping systems. When inoculating *Medicago truncatula* with three *Rhizoglomus* species, *R. intraradices*/*irregulare*, *R. aggregatum* and *R. custos*, Kiers et al. [103] observed that host plants were able to detect, discriminate and select the most beneficial AMF symbionts. Of the three species, *R. intraradices*/*irregulare* was the most outstanding, delivering the highest quantities of nutrients to *M. truncatula* and acquiring the highest amount of carbohydrates itself. Following double or triple combinations of *Funneliformis mosseae*, *R. intraradices*/*irregulare* and *Entrophospora claroidea* (all belonging to the Order Glomerales), Jansa et al. [36] obtained similar results as in our study: no additional crop growth benefits to *M. truncatula* were observed following combinations, compared to the inoculation of *F. mosseae* alone. When *A. porrum* was inoculated with the same combinations, however, plant biomass was lower than inoculation with *F. mosseae* alone. These studies consequently demonstrate the complexity of the interactions and associations between host plants and AMF, as well as between AMF species/strains on the same plant. Maherali and Klironomos [104], working with *Plantago lanceolata* showed that host plant growth benefits were greater when combinations of AMF included species from different families, compared with combinations of species from within a single family. This concept was further supported by Thonar et al. [105] and Yang et al. [40] who indicated that different AMF families have complementary functional capacities in favor of the plant hosts. A number of recorded species of AMF have previously shown beneficial effects on host plants, such as *Dioscorea rotundata*, e.g., *Entrophospora etunicata*, *E. claroidea*, *A. scrobiculata* and *A. spinosa* [44]. Many other recorded species have, to date, rarely been cultivated and so their potential benefits remain unknown, for coffee and for any other hosts. From the 35 AMF species detected and the four most abundant and frequently occurring AMF species in our study, just two species multiplied efficiently for inoculum production.

Following inoculation in the current study, AM fungal root colonization for *R. variabile* or the combined inoculation was similar (~85%) after 135 days, while colonization for *N. plukenetiae* alone was lower (68%). Similarly, Säle et al. [100] also observed that AMF isolates with the highest root colonization rates provided the greatest growth benefits. Dumbrell et al. [106], further concluded that AMF species with high root colonization rates are more effective at creating symbiotic associations and providing more benefits to host plants than slower-growing fungi. Both *R. variabile* and *N. plukenetiae* belong to major clades of the Glomeraceae, which are known for their high root colonization capacity [100,107,108].

Coffee inoculation with AMF resulted in exceptional root length increases, of up to 84%, which is higher than previously reported for coffee under greenhouse conditions [47,48,55,56,104,109]. Such levels of improvement may be an indication of the high AMF dependency of coffee plants, especially in marginal soils with low fertility [45,48,53,69,110,111].

When considering which AMF isolates should be applied, or developed as a product, the high colonization rate is an important criterion, as is the speed of colonization and consequent nutrient assimilation. Voříšková et al. [112], for example, observed the dominance of *R. irregulare* (92%) in combined inoculations on *M. truncatula*. Its high capacity to colonize roots enables it to rapidly occupy root niches and possibly exclude other AMF species [43,113].

Our results on N, P and K assimilation appeared dependent on the AMF association. *N. plukenetiae* had a lower efficiency of P assimilation than *R. variabile*. Phosphorus can be directly uptaken by the plant through the mycorrhizal networks [114,115,116]. Andrade et al. [55], reported an average three-fold higher P assimilation in mycorrhizal coffee plantlets initially inoculated with an AMF consortium, reflecting results for *N. plukenetiae* in our study. Smith et al. [117], also showed that *R. intraradices*/*irregulare* transferred about 100% of the assimilated P in three plant species, but that *Funneliformis caledonius* and *Gigaspora rosea* delivered much less. However, the mycorrhizal symbiosis can also result in the inactivation of direct P uptake by the roots [117,118,119,120]. In other studies, using the split-root system, in which different root areas of the same plant were colonized by different AMF, host plants were able to discriminate between the fungi, delivering Carbon (C) preferentially to the most beneficial AMF species [22,26,121,122,123]. In our study, the lowest quantities of P were recorded in plants in the dual inoculation treatment with *R. variabile* and *N. plukenetiae*, indicating that either *N. plukenetiae* interfered in the transfer of P to the plants, or that there was competition between the two species for P acquisition. In this context, Jansa et al. [36] reported that certain combinations of AMF strains (consortia) reduced the P content in *A. porrum* leaves when compared to single inoculations. In contrast, Crossay et al. [34] found that combined inoculations with species from different AMF families increased shoot P concentrations, compared to single inoculations with specific AMF species.

Conversely, to P acquisition, N content was superior following inoculation with *R. variabile* alone or combined with *N. plukenetiae* than with *N. plukenetiae* alone. Lower N concentrations in the *N. plukenetiae* treatment were closely related to the lower biomass of the coffee roots, and the lower AMF colonization of the roots when compared to the single or dual inoculations with *R. variabile*. Numerous studies have shown that AMFs are able to absorb large amounts of inorganic nitrogen as nitrate-N or ammonium-N [124,125,126,127,128,129] and at least partially transfer it to the plant hosts [25,27,100,130,131,132,133]. Although N is much more easily assimilated by plants than P, N limitation in ecosystems may lead to increased levels of N plant uptake through the mycorrhizal network [124,134] even though some studies have indicated that N assimilation through AMF is not important for the plant [135]. Nevertheless, as shown in our study, N assimilation can be affected by specific AMF species, and that *R. intraradices*/*irregulare* is superior to other Glomeraceae species (*Glomus*/*Simiglomus hoi*) [130]. Our findings further confirm, therefore, that although the AMF symbiosis is not species-specific, the compatibility and efficiency between the plant host and its AM fungal associates may largely depend on the specific plant-fungal association [136]. The respective differences in N contents for coffee plants inoculated with *N. pluneketiae* and *R. variabile*, were similarly reflected in photosynthetic activity and leaf chlorophyll contents (48.9 vs. 59.8 SPAD, respectively), which may have limited the transfer of C to the fungi. Various studies have previously demonstrated the strong correlation between leaf N contents and photosynthesis [137,138,139].

As for P, AMF can also increase the K availability for host plants, which can otherwise be low due to its strong mineral absorption [43,140,141,142,143]. In our study, treatment with AMF led to over four times higher K contents in coffee leaves (42.9–77.6 mg K kg^−1^) than the non-mycorrhizal control (17.5 mg kg^−1^), which is much higher than Andrade et al. [55] reported for mycorrhizal coffee seedlings. Similar to our study, Garcia and Zimmermann [142] and Olsson et al. [144,145], also found a clear relationship between the P and K leaf contents during the AM symbiosis.

## 5. Conclusions

The current study clearly shows the high growth-promoting and biofertilizing potential of two recently described AMFs for coffee seedlings. Both fungi are indigenous to Peruvian coffee plantations but are not restricted to South American ecosystems. Remarkably, *Rhizoglomus variabile* is now the sixth known *Rhizoglomus* species, after *R. intraradices*, *R. irregulare*, *R. fasciculatum, R. clarum* and *R. invermaium*, demonstrating the huge potential to promote crop growth. The potential benefits of these closely related species, consequently, demand greater attention for their benefit to agriculture. Similarly, *Nanoglomus* also appears to be a genus of interest towards enhancing plant growth and agronomic performance, but the combination of both studied species belonging to the same AMF family had no synergistic effects for the coffee plants. Our study further demonstrates the importance of identifying the most suitable species/strains. Nevertheless, multiple species applications might be most beneficial either for the crop plants or for the whole plant-soil system, especially when AMF strains from other AMF families could be integrated. The results indicate an initial step towards the use of such AMF strains as a profitable and environmentally sustainable strategy for the establishment of coffee plantations, both in traditional and sustainable modern low-input agro-forestry systems. Further steps towards the commercial production of AMF, based on *R. variabile*, would be to evaluate other relevant species from the Glomeromycetes, alone and in combination with *R. variabile*, and to select the most beneficial combinations for coffee production in the Peruvian mountains and beyond.

## Figures and Tables

**Table 1:** Geographic coordinates of coffee plantations in San Martín State (Peru) under AMF diversity study.

Province	Location	Area (ha)	Age	Associated Crops	Cultivar	Fertilizer	Geographic Coordinates		Altitude (m)
							X	Y	
Lamas	Alto Palmiche (LA-1)	2	6	*Inga edulis, Eucalyptus* sp.	Pache, Bourbon	Organic	6°20′3.10″	76°35′58.96″	978
	Pamashto (LA-2)	1	10	*Inga edulis*	Catimor	Organic	6°21′8.59″	76°32′15.66″	851
	Pueblo Nuevo (LA-3)	6	10	*Inga edulis*	Catimor, Bourbon	Organic	6°19′5.68″	76°42′26.41″	1088
El Dorado	Palestina (ED-1)	3	8	*Inga edulis, Citrus* sp.	Catimor, Pache	Organic	6°27′46.32″	76°49′18.70″	745
	Requena (ED-2)	1.5	3	--	Catimor, Pache	Organic	6°31′1.30″	76°45′38.38″	468
	San Juan de Talliquihui (ED-3)	1	7	*Inga edulis, Mangifera indica*	Catimor, Caturra	Organic	6°37′44.26″	76°36′16.96″	602
San Martín	Santa Rosa de Huayali (SM-1)	0.75	4	*Inga edulis, Persea* sp.	Catimor	Organic	6°44′32.12″	76° 9′11.51″	740
	Nuevo Lamas (SM-2)	2	6	*Inga edulis*	Catimo, Pache	Organic	6°36′6.67″	76°11′56.36″	973
	Nuevo Porvenir (SM-3)	2	6	*Inga edulis*	Caturra, Pache	Organic	6°45′40.66″	76° 7′20.22″	782
Moyobamba	Barranquita (MO-1)	6	4	*Inga edulis, Musa* sp.	Catimor, Caturra	Organic	6°10′20.91″	76°53′47.71″	1054
	Palmeras (MO-2)	2	4	*Inga edulis, Cedrela* sp.	Catimor, Caturra	NPK (20-7-10)	6° 6′25.25″	77° 1′33.53″	945
	Cocha Negra (MO-3)	3	8	*Inga edulis, Citrus* sp.	Catimor, Caturra	Organic	6°32′37.07″	76°54′48.36″	860

**Table 2:** Arbuscular mycorrhizal fungi species identified from twelve coffee plantations in San Martín State, Peru.

Provinces	El Dorado			Lamas			San Martín			Moyobamba			Occurrence (%)
AMF Species	ED-1	ED-2	ED-3	LA-1	LA-2	LA-3	SM-1	SM-2	SM-3	MO-1	MO-2	MO-3	
*Glomus microcarpum*	++	++	++	+++	++	+++	+++	+	+	++	++	+++	100
*Nanoglomus plukenetiae*	+++	+++	+	++	+	++	++	+	++	+	+++	++	100
*Rhizoglomus variabile*	+	+++	+	++	+++	+++	++	++	+++	+	++	+++	100
*Acaulospora mellea*	++	-	++	+++	+++	+++	++	++	+++	+	+	+++	92
*Glomus brohulti*	+	-	-	+++	+++	+++	++	+++	+++	+	+++	+++	83
*A. spinosissima*	+	+	-	+	+	+	-	+	+	+	-	-	67
*Entrophospora etunicata*	-	+	++	-	+++	+	+++	++	+++	-	+	-	67
*Entrophospora claroidea*	-	-	++	-	-	+++	++	++	++	+	+	-	58
*Glomus macrocarpum*	++	++	-	-	-	++	+++	+	+	-	+	-	58
*Funneliglomus sanmartinense*	+	-	+	++	-	-	-	-	-	+	+	+	50
*A. laevis*	-	-	-	+	-	++	-	+	-	++	+	-	42
*A. scrobiculata*	+	-	-	-	++	+	-	-	-	+	-	+	42
*Rhizoglomus fasciculatum*	-	-	+	-	-	+	+	+++	+	-	-	-	42
*Ambispora* sp. resembling *A. gerdemannii*	-	-	-	-	+	+	-	-	-	-	+	+	33
*Diversispora spurca*	-	-	-	++	-	++	+	+	-	-	-	-	33
*A. lacunosa*	-	-	-	-	+	-	-	-	-	+	+	-	25
*Dominikia* sp.1	-	-	-	-	-	++	-	-	-	+	+	-	25
*Gigaspora candida*	-	-	+	-	-	-	-	-	-	+	-	+	25
*Glomus* sp. 2	-	-	+	-	-	-	-	+	-	-	+	-	25
*Sclerocystis sinuosa*	-	-	-	-	-	++	-	++	-	++	-	-	25
*Glomus* sp. resembling *G. spinuliferum*	-	-	-	+	-	-	-	+	-	-	-	-	17
*Glomus* sp. 3	-	-	-	-	-	-	+	-	-	-	-	+	17
*Glomus* sp. 4	-	-	-	-	-	-	+	-	-	-	-	+	17
*A. spinosa*	-	-	+	-	-	+	-	-	-	-	-	-	17
*Acaulospora* sp. resembling *A. pustulata*	-	-	-	-	-	-	+	-	-	-	-	-	8
*A. herrerae*	-	-	-	-	-	-	-	-	-	+	-	-	8
*A. excavata*	-	-	-	-	-	-	-	+	-	-	-	-	8
*Dominikia* sp. 2	-	-	-	-	-	-	-	+	-	-	-	-	8
*Glomus crenatum*	-	-	-	-	+	-	-	-	-	-	-	-	8
*Glomus* sp. 1	-	-	-	-	+	-	-	-	-	-	-	-	8
*Glomus* sp. 5	-	-	-	-	-	+	-	-	-	-	-	-	8
*Rhizoglomus microaggregatum*	-	-	-	-	+	-	-	-	-	-	-	-	8
*Sclerocystis rubiformis*	-	-	-	-	-	-	-	+	-	-	-	-	8
*Sclerocystis* sp. 1	-	-	-	-	-	-	+	-	-	-	-	-	8
*Sieverdingia tortuosa*	-	-	-	-	-	-	+	-	-	-	-	-	8
AMF species richness/site	9	6	11	10	13	17	15	18	10	15	14	11	
AMF species richness/province		14			24			23			21		

Location number, 1–2–3. -: absent (0 spores/g); +: 1–2 spores/g; ++: 3–5 spores/g; +++: >6 spores/g. Bold indicates abundant (>3 spores/g) and ubiquitous (i.e., detected in all soil samples) AMF species in the field samples.

**Table 3:** Dominant sporulating arbuscular mycorrhizal fungi species isolated after culturing for three months in the screenhouse.

Provinces	El Dorado			Lamas			San Martín			Moyobamba			Occurrence (%)
AMF Species	ED-1	ED-2	ED-3	LA-1	LA-2	LA-3	SM-1	SM-2	SM-3	MO-1	MO-2	MO-3	
*A. mellea*	+	+	++	+++	++	+++	++	+	++	-	-	-	75
*R. variabile*	++	+++	++	++	+++	++	++	++	+++	+	++	+++	100
*N. plukenetiae*	+	++	+	++	+	+++	++	+	++	+	++	++	100
*G. microcarpum*	-	-	-	+	++	++	+++	+	++	++	+++	-	58

Location number, 1–2–3. -: absent (0 spore/g); +: 1–2 spores/g; ++: 3–5 spores/g; +++: >6 spores/g. Species in bold indicate abundant and ubiquitous AMF species in trap cultures.

**Table 4:** Impact of AMF single and dual inoculation on *Coffea arabica* growth and physiology after 135 days.

Treatment	Shoot Fresh Matter (g)	Roots Fresh Matter (g)	Shoot Dry Matter (g)	Roots Dry Matter (g)	Chlorophyll Content (SPAD)	Leaf Area (cm^2^)
Control (Ctr)	3.0 ^c^ ± 0.5	0.8 ^c^ ± 0.2	0.77 ^c^ ± 0.01	0.21 ^c^ ± 0.01	27.4 ^c^ ± 0.28	155 ^c^ ± 6.01
*R. variable* (Rv)	15.3 ^a^ ± 2.4	7.4 ^a^ ± 1.2	3.58 ^a^± 0.04	1.81 ^a^ ± 0.05	59.8 ^a^ ± 0.50	691 ^a^ ± 17.08
*N. plukenetiae* (Np)	9.7 ^b^ ± 1.7	4.4 ^b^ ± 1.1	2.25 ^b^ ± 0.03	1.04 ^b^ ± 0.06	48.9 ^b^ ± 0.58	434 ^b^ ± 13.35
Rv+Np	15.9 ^a^ ± 2.1	7.8 ^a^ ± 1.0	3.63 ^a^ ± 0.03	1.77 ^a^ ± 0.02	60.3 ^a^ ± 0.26	709 ^a^ ± 14.60
*p* and F-Value						
	*p* < 0.0001	*p* < 0.0001	*p* < 0.0001	*p* < 0.0001	*p* < 0.0001	*p* < 0.0001
	F = 337.1	F = 373.7	F = 2130.0	F = 303.5	F = 1288.4	F = 376.63

Means ± standard deviation of 34 replicates. Treatments with the same letter do not significantly differ between each other (ANOVA followed by Tukey’s HSD; *p* < 0.05)

## Data Availability

Data are contained within the article.
